# Endophytic *Isaria javanica* pf185 Persists after Spraying and Controls *Myzus persicae* (Hemiptera: Aphididae) and *Colletotrichum acutatum* (Glomerellales: Glomerellaceae) in Pepper

**DOI:** 10.3390/insects12070631

**Published:** 2021-07-12

**Authors:** Roland Bocco, Moran Lee, Dayeon Kim, Seongho Ahn, Jin-Woo Park, Sang-Yeob Lee, Ji-Hee Han

**Affiliations:** 1 Agricultural Microbiology Division, National Institute of Agricultural Sciences, RDA, Wanju 565-851, Korea; rolandbocco@gmail.com (R.B.); ahfks2@korea.kr (M.L.); dayeon413@korea.kr (D.K.); ash0510@korea.kr (S.A.); jinwoopark@korea.kr (J.-w.P.); 2 Department of Agriculture and Natural Resources (ANR), UC Davis, Cooperative Extension, 2156, Sierra Way, Ste C, San Luis Obispo, CA 93401, USA; 3 The Korea Agro-Materials Research Organization (KARO), 703, Farmer’s Building 92, Suseong-ro, Paldal-gu, Suwon-si 16432, Korea; lupfire@naver.com; 4 Planning and Coordination Division, National Institute of Agricultural Sciences, RDA, Wanju 565-851, Korea

**Keywords:** insecticidal, antifungal, hemocytometer, isolate, aphid mortality, leaf damage percent

## Abstract

**Simple Summary:**

The green peach aphid (*Myzus persicae*) and the phytopathogenic fungus *Colletotrichum acutatum* cause significant losses in a wide variety of crops. To efficiently protect their crops, farmers use chemical pesticides, but this kind of practice is not sustainable because of its negative effects on the environment. This study suggests an environmentally friendly method such as the use of the endophytic *Isaria javanica* pf185 in pepper plants. Suspension of the endophytic fungus (EF) was sprayed on plants under cage while those same leaves were sampled and assessed under laboratory conditions. The EF can both penetrate inside the leaf tissues and survive on the surface of the leaf after five weeks. The suspension showed an evident insecticidal efficiency against *M. persicae* and a lower one against *C. acutatum*. Therefore, its antifungal efficiency against *C. acutatum* was not correlated with weather patterns. Authors recommend *I. javanica* pf185 as a potential biocontrol agent against *M. persicae* and *C. acutatum*.

**Abstract:**

This study endeavored to sustainably control aphids and anthracnose after spraying endophytic *Isaria javanica* pf185 under field conditions. Under two different tents; one batch of seedlings was sprayed with a 10^7^ conidia/mL *I. javanica* pf185 suspension; while another was sprayed with 0.05% Tween 80^®^ in distilled water. Six leaf discs from the top; middle; and bottom part of the plant canopy were weekly collected and placed on moistened filter paper in a Petri dish for insecticidal and antifungal bioassays against *Myzus persicae* and *Colletotrichum acutatum*. Differences were noticed from the 18th day after spraying with mortality (86.67 ± 0.57% versus 36.67 ± 0.64%) and leaf damage (13.45 ± 0.03% versus 41.18 ± 0.06%) on fungus-treated and controlled, respectively. The corrected insecticidal efficacy was 20.43, 39.82, 72.32, 66.43 and 70.04%, while the corrected fungicidal efficacy was 26.07, 38.01, 53.35, 29.08 and 41.81% during five successive weeks. A positive correlation was evident between insecticidal efficacy and relative humidity (r^2^ = 0.620) and temperature (r^2^ = 0.424), respectively. No correlation was found between antifungal activity and relative humidity (r^2^ = 0.061) and temperature (r^2^ = 0), respectively. The entomopathogenic fungus survived on leaf surface area and in tissues after spraying.

## 1. Introduction

Cultivated pepper, *Capsicum annuum* L., consists of phenotypically diverse plant species grown all over the world [1]. This plant is formerly used in traditional and modern medicine for its powerful properties to cure many ailments [2,3]. It is a source of financial income for farmers in many countries when sold as a vegetable [4]. Like many vegetable crops, pepper production is dealing with biotic constraints, including injury by insect pests such as the green peach aphid (*Myzus persicae*, Hemiptera: Aphididae) and diseases such as anthracnose (*Colletotrichum acutatum*).

The aphid species *M. persicae* is reported as highly polyphagous, with a host range of over 400 species in 40 different plant families, including many economically important crop plants [5]. Infested leaves become curly at first, then turn yellow, followed by deformation and drying; while the fruits are smaller, lack of peer appearance and taste [6,7]. Later, a dirty black film is visible, which consists of a sooty mold that grows on the aphids that excrete honeydew. Galls might also develop on the leaves due to aphid infestations. The increasing number of galls and the sooty mold progressively reduces photosynthetic performance of leaves by affecting chlorophyll, stomatal conductance, and carotenoid contents [8,9]. Sooty mold comprises saprophytic fungi with facultative associates of honeydew-producing Hemiptera, particularly in scale insects and aphids [10]. They form black-colored colonies on leaf surfaces that have received honeydew. These fungi include taxa from five families of Ascomycota, as Antennularielliaceae, Capnodiaceae, Chaetothyriaceae, Euantennariaceae and Metacapnodiaceae (Order Dothideales) [11].

Aphids also transmit viruses that can cause yield loss of 75–82%, affect plant height, the number of fruits per plant, fruit length, the number of seeds per fruit, and fruit weight [12]. For example, Singh [13] reported 35.4, 35.7, and 41.9% overall loss in the yield of knol-khol (*Brassica caulorapa*), radish (*Raphanus sativus*), and cauliflower (*Brassica oleracea* var. botrytis) crop when affected by aphids, while Reetsang [14] reported that yield loss increased to over 70% in cabbage when any insecticide was not applied.

The anthracnose causing agent *C. acutatum* is one of major economically damaging disease plant pathogens that infects a wide range of hosts that includes cereals, legumes, vegetables, perennial crops, and tree fruits [15,16]. Among these hosts, *C*. *annuum* is severely infected by *C. acutatum*, which may cause yield losses of up to 100% in the absence of any fungicide application [17]. Characteristic symptoms on fruit include sunken necrotic tissues, with concentric rings of acervuli, ultimately reducing fruit marketability [18,19,20,21].

Farmers use expensive chemicals to protect their fruit production avoiding significant losses originated by *M. persicae* and *C. acutatum* [22,23]. However, these pesticides not only attack the targeted pest and disease; but also have a side effect on nearby ecosystems [24]. Nowadays, residues of pesticides can be found in several varieties of everyday foods and beverages including cooked meals, water, wine, fruit juices, refreshments, and animal feeds [25,26,27]. Moreover, insecticide resistance is building up and is becoming a challenge for both insect pest and plant pathogen management, limiting the use of some chemical control tools for competitive crop yields [22,28,29]. Several research projects have been undertaken for over two decades, for developing an ecologically sound protection of vegetable crops, using integrated pest management (IPM) methods as host plant resistance, and biological control for a sustainable crop protection [30].

Biological control methods include the use of insect pathogens like *Beauveria bassiana*, *Metarhizium anisopliae*, *M. rileyi*, *Verticillium lecanii*, *Isaria* spp., and others that have been found promising in the control of several important agricultural pests [31]. For instance, the isolates *Isaria javanica* pf212 and *I. javanica* pf185 were found to be efficient for the control of *M*. *persicae*, *C*. *gloeosporioides* and *Phytophthora capsici* under laboratory conditions [32]. Research showing the colonization of the leaf tissue by the two isolates *I. javanica* pf212 and *I. javanica* pf185 has yet to be fully explored.

This five-week experiment aims to evaluate (a) the insecticidal and fungicidal activities of the entomopathogenic fungus *I. javanica* pf185 to protect pepper plants against *M. persicae* and (b)*. acutatum* and (c) its endophytic and ectophytic presence after spraying under field conditions to confirm in vitro findings of Kang et al. [32].

## 2. Materials and Methods

### 2.1. Experimental Sites

**Figure 1:**
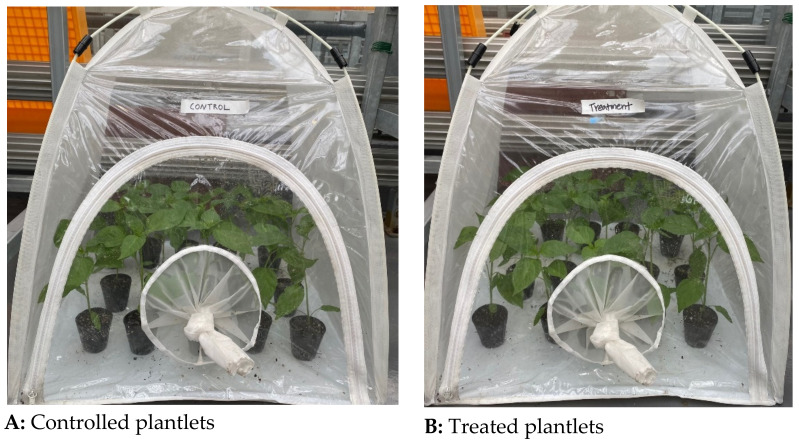
Controlled plantlets (**A**) and fungus-treated plantlets (**B**) under tents.

The experiment was conducted in five successive weeks on individual potted plants settled under insect rearing tents (60 × 60 × 60 cm) at the National Institute of Agricultural Sciences (RDA) in Jeonju (35°49′ N, 127°09′ E, and 53.5 m sea level) located in the southwestern part of the Republic of Korea. To allow least variation between the two tents after spraying, they were laid about one meter from each other. Under these conditions, the average number of leaves per plantlet and its height could not vary between tents during the experiment (Figure 1).

### 2.2. Insects and Plants

Neonates of *M. persicae* were obtained from the entomology unit of RDA and reared on pepper plants. For that, pepper seeds with pericarp previously sterilized in 70% ethanol for 3 min were dried under a laminar flow cabinet (CHC LAB; Model No. CLB-202D, CHC LAB Co., Ltd.; 139, Techno 2-ro, Yuseong-gu, Daejeon, South Korea). Then, they were individually planted in pots (1.5 L) that contained the commercial soil medium (Baroker Seoul Bio Co., Ltd., Eumseong, Korea) that was previously sterilized at 121 °C for an hour [33].

### 2.3. Conidial Suspensions

Two fungal isolates were used: the endophytic *I. javanica* pf185 (entomopathogenic fungus) obtained from the Korea Agricultural Culture Collection (KACC), registered as KACC93241P and the plant parasitic fungus *C. acutatum* (KACC 40689). They were isolated from soil samples collected in South Korea locations 36°49′ N, 126°36′ E, and 37°82′ N, 129°04′ E, respectively. Based on earlier studies by Kang et al. [32], the isolate *I. javanica* pf185 appears to have microbial control potential against several pests, while the pathogenic *C. acutatum* was identified as the cause of wide ranges of damages to pepper crops in Korea. They were cultivated in an incubator (25 ± 1 °C, 98 ± 2% RH; and 12: 12 L:D) on potato dextrose agar (PDA) medium for 14 days prior to use. Conidia were harvested and suspended in 0.05% Tween 80^®^ solution made with sterilized water and vortexed for 3 min to get a homogenous mixture. This was then filtered through four screens made of cheesecloth to remove hyphae and other particles [34]. Through a hemocytometer, concentrations were adjusted to 10^7^ and 10^6^ conidia of *I. javanica* pf185 and *C. acutatum* per milliliter (mL) suspensions, respectively [34]. A 0.05% Tween 80 solution (free of conidia) in sterile water was used as control and then vortexed for 3 min.

### 2.4. Experimental Set-Up

The bioassays were performed in a growth chamber (Bionex Multi-Room Incubator VS-1203PFC-L, VISION SCIENTIFIC CO.; LTD., 823 Toprip Dong Yuseong-Gu Daejeon Si, South Korea) at 25 ± 1 °C, 75 ± 10% relative humidity (RH) and 12:12 L: D. At the beginning of the experiment, 50 seeds were individually planted in different pots in a greenhouse in the RDA and protected from insects and diseases using screened tents made with fine mesh (~0.1 × 0.1 mm) ([35] with a few modifications). Three weeks after planting, two sets of 15 uniformly sized, healthy-looking plants were transferred into two separate tents under a plastic house. The first batch designed as control was sprayed with a solution of Tween 80^®^, while the second one designed as fungus-treated was sprayed with a suspension of *I. javanica* pf185. Potted plants were watered daily with a trigger watering can at the root collar, avoiding contact with the leaves.

### 2.5. Seedling Inoculation and Management

Two treatments were deployed: (1) a tent with potted pepper seedlings individually sprayed with 10 mL of endophytic fungus suspension; and (2) a tent with potted pepper seedlings individually sprayed with the control solution through test tubes and nozzles (Trigger Sprayer, 7–1/4, Tolco Corporation, 1920 Linwood Avenue Toledo, OH, USA).

In both tents, the plants were sprayed once in four weeks after planting. Plants were fertilized according to the Korean standard recommendations of 225, 64, and 101 kg/ha of N, P, and K, respectively [36]. The dose of the nutrient supplied per pot (1.5 L) was 0.2 g, 0.06 g, and 0.09 g of N, P and K, respectively (Bocco, R. personal communication).

To promote a good adhesion of the spores of the entomopathogenic fungus to the leaf blade of the leaves, no sampling was implemented on the day of spraying. During week zero of the experiment where the spraying had taken place, leaves were sampled the day after spraying while the fungal isolation from leaves started three days after spraying. In the following assessments from week 1 to week 4, no further spraying was done.

Data were collected five times from each treatment at weekly intervals after the single conidial sprays to evaluate the persistence of the entomopathogenic fungus.

Temperature and relative humidity patterns were recorded every 30 min from one week before both the suspension preparation and the first leaf sampling to the end of assessments using an Onset HOBO (U12 Temp/RH/2 External Channel Logger, Onset Computer Corporation, 470 MacArthur Boulevard, Bourne, MA, USA).

### 2.6. Leaf Sampling for I. Javanica pf185 Biocontrol Assessments and its Persistence via Isolation

Once a week, three leaves were sampled from three fungus-treated plants in a-batch and three other leaves from the control ones. From week 0, leaves were bioassayed during five successive weeks (W0–W4). The leaves were early sampled in the morning and kept at 4 °C in a cooler containing ice packs to prevent desiccation. With a cork borer, 2 cm-diameter leaf discs were cut from these leaf samples and used for experimental purposes in the laboratory. A total of 12 leaf discs cut from six leaves collected at the top, center, and bottom of each individual plant were used weekly to test both the insecticidal and fungicidal action of the endophytic fungus. Six leaf discs per single plant were used for each endophytic study via isolation.

### 2.7. Effect of I. Javanica pf185 on M. Persicae

**Figure 2:**
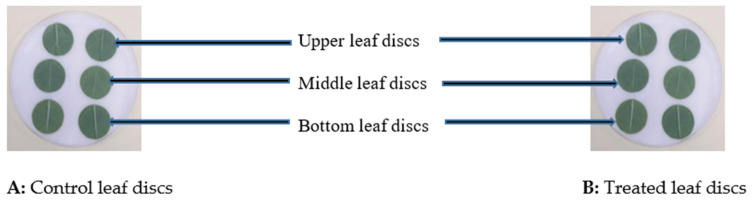
Assessment on control (**A**) and fungus-treated (**B**) leaves sampled from a single plant.

On each assessed plant, three pairs of 2 cm-diameter leaf discs were placed on filter paper moistened with 10 mL sterile distilled water in a 9 cm-diameter Petri dish (Figure 2). Solid organic debris was carefully removed from the leaf disc surfaces using a camel’s hair brush. Five neonate aphids were placed on each leaf disc using the same brush that was disinfected with 70% alcohol to avoid any contamination by microorganisms. After aphid inoculation, the Petri dishes were incubated at 25 ± 1 °C Temp; 98 ± 2% RH; 12:12 L:D. Number of dead aphids (mortality) per leaf disc was recorded daily from the 3rd to the 6th day after exposure of neonate aphids. Daily, dead aphids were individually mounted on a slide in lactophenol blue solution and observed microscopically (LEICA DMRE) for fungal growth. Weekly, the experiment lasted 4 days (3rd to 6th days). The experiment was repeated for five weeks. Similarly, at the end of each experiment, every single aphid that remained alive was microscopically observed to determine the presence of entomopathogenic fungus on its body or internal structure. Those living with fungal mycelium were considered infected, while the rest were recorded as alive or non-infected.

### 2.8. Effect of I. Javanica pf185 on C. Acutatum

The method used in this experiment was identical to the one described in Section 2.6. The difference was that the aphids were replaced by the inoculum of the phytopathogenic *C. acutatum*. Wounds (0.5 ± 1 mm diameter each) were made in the middle of each leaf disc (1 wound/leaf disc). Then, 10^6^ conidia/mL in 5 μL suspension of *C. acutatum* were deposited on each wound using a micropipette and allowed to dry under a laminar flow cabinet. The negative control treatment consisted of leaf discs that received 5 µL of distilled water each. Through a microscope LEICA MZ 125; the individual diameter of each wound per leaf disc was measured. All Petri dishes that contained inoculated leaf disks and control ones were covered and placed in an incubator (25 ± 1 °C, 98 ± 2% RH, 12:12 L:D) to be observed from the 3rd to the 6th day (Figure 2).

### 2.9. Examining the Endophytic Presence of I. Javanica pf185

To achieve the goal of determining the penetration and persistence of the fungus in plant tissues; the following successive operations were conducted during five successive weeks.

(a) Samples of leaf discs were washed under tap water to remove dust and rinsed three times with distilled water. To suppress microorganisms on the leaf surface, the samples were surface-sterilized with 70% ethanol for 30–60 s, followed by a 5% sodium hypochlorite bath for 3 min. The leaves were finally rinsed five times with sterile distilled water and left on sterile tissue paper [37] to dry. Three dried leaf discs from the top, medium, and bottom parts of plants (i.e., nine leaf discs) were each cut using a 0.8 cm-diameter cork borer. Then, the leaf discs were cut with a sterile blade into 1 × 20 mm segments to avoid inoculating new microorganisms. Each leaf segment from the same plant was placed onto a PDA medium supplemented with 500 mg/l chloramphenicol (PDA-C) to suppress bacterial growth [38]. To confirm success of the sterilization, the final rinse with 50 µL distilled water was placed on a PDA medium as a negative control. All plates were incubated in the dark at 25 °C and daily examined for four weeks because growth rate is not identical between taxonomic groups. The success of the leaf piece sterilization was confirmed on Control PDA plates from which no fungal or bacterial growth was observed.
(b) Fungal spores that grew from sterilized leaf surface were extracted from 4 week-old PDA plates by adding 10 mL sterile 0.05% Tween 80^®^ solution, and the spores were filtered through four screens made of cheesecloth to remove hyphae and other particles. The mixture was then vortexed for 3 min to get a homogenous mixture of spores. To start a single spore culture of the different harvested species, using a hemocytometer, each suspension was adjusted to 10^3^/mL as described by Han et al. [34]. Then, the underside of each Petri dish containing fresh PDA was marked at 1 cm × 1 cm with a pen. Through a micropipette 1 μL drop of the suspension was placed above the PDA at the middle of each square and allowed to grow. Between 80–100 drops were placed on each PDA plate weekly to identify the maximum of taxonomic groups present.
(c) The developed fungal colonies were purified through successive transplanting and single spore techniques. After slide culturing, the purified endophytic fungi were identified according to the fungal morphological and micromorphological characteristics as described [39,40,41]. At the end, only the white colonies obtained had been considered for this experiment to eliminate ones that did not resemble *I. javanica* pf185, which is originally white. The different taxonomic groups isolated were stored at 5 °C in a solution of 40% glycerol and water. Similarly, the endophytic colonization frequency (Endo CF) of the different fungi was calculated individually for the collected samples.

(1) $$Endo\;CF=\frac{Amount\;of\;identical\;white\;colonies}{Amount\;of\;white\;colonies}\times 100$$

### 2.10. Weekly Ectophytic Presence of I. Javanica pf185 after Spraying

To evaluate the persistence of fungus outside leaf tissues of sprayed plants, successive and complementary studies were conducted simultaneously with the endophytic study.

(a) From leaf samples collected, respectively, at the top, middle, and bottom of each plant, 0.5 cm-diameter leaf discs were cut (three discs from each plant part). The discs were then placed in a tube that contained five milliliters of 0.05% Tween 80^®^ solution and vortexed for 1 min to promote detachment of the adhered spores. Ten milliliters of this solution were transferred onto PDA plates (three plates per sample), which were sealed and incubated at 25 °C. Three days after incubation, the average number of colony-forming units (CFUs) was counted and estimated as CFUs/mm^2^ per plant. All colonies of *I. javanica* were microscopically observed and counted to estimate ectophytic colonization frequency (*Ecto CF*) on the leaf surface per week using the formula:
  (2) $$Ecto\;I.\;javanica=\frac{Weekly\;amount\;of\;I.\;javanica}{Amount\;of\;I.\;javanica\;colonies\;recorded\;for\;five\;weeks}\times 100$$
(b) Then, the spore concentration of the same leaf washed solution was determined through a hemocytometer. The average spore concentration of mixture from each sterilized leaf surface was estimated in conidia/mm^3^ with three replications from top, middle, and bottom canopy per single plant. All CFUs present on the medium were counted.
(c) Finally, the three leaf discs were vortexed, removed from each tube and placed on filter paper moistened with distilled water in Petri dishes and allowed to dry under a laminar cabinet. After drying, the number of spores remaining adhered to the leaf blade was carefully counted using a stereoscopic electric microscope (LEICA DMRE). By leaf disc, the number of spores in three random microscopic fields were counted. By plant, nine microscopic fields or observations were taken into account per week. The average number of spores adhered to each leaf was estimated in conidia/ mm^2^. All the spores observed under the microscope were recorded.

### 2.11. Processing of Data

For estimating *I. javanica* pf185 insecticidal and fungicidal activities, mean percentages of mortality and injury increase were calculated daily and weekly on fungus-treated and control plants. Six leaf discs were recorded per single plant, i.e., 18 leaf discs by treatment per day, but the average values per treatment and week were estimated by combining 18 leaf discs per plantlet per day for five successive weeks.

Since only three Petri dishes or plants were used per treatment (control and fungus-treated) as experimental units with six leaf discs sampled from the top, middle and bottom of each plant; daily adjusted average values were estimated by plant for aphid mortality and leaf wound diameter.

The daily (day 3–6) percent of mortality (*Dy_Mt*) was estimated using the daily number of dead aphids according to the following formula:

(3) $$Dy\_Mt=\overset{x}{\underset{i=0}{\sum }}\frac{\left(Alive\;D0-Alive\;Dy\right)}{Alive\;D0}\times 100$$

where *Dy_Mt* is the mortality on any given day, *Alive Dy* is the number of live aphids recorded on that day; *Alive D*0 is the number of live aphids exposed per leaf disc.

The weekly (week 0–4) percent mortality (*Wt_Mt*) was estimated using the daily number of dead aphids according to the following formula:

(4) $$Wt\_Mt=\overset{x}{\underset{i=0}{\sum }}\frac{\left(Alive\;D0-Alive\;Wt\right)}{Alive\;D0}\times 100$$

where *Wt_Mt* is the mortality at any week, *Alive Wt* is the number of live aphids recorded on the 6th day in any given week, *Alive D0* is the number of live aphids exposed per leaf disc.

The percent of increasing in leaf damage (*ILD*) was obtained by measuring the diameter of each wound every day in order to estimate its growth percentage using the following formula:

(5) $$Dy_{ILD}=\frac{\left(Diameter\;Dy-Diameter\;D0\right)}{Diameter\;D0}\times 100$$

where *Dy_ILD_* is the increasing in leaf damage on any given day; *Damage Dy* is the leaf wound diameter increase recorded on a given day; *Damage D0* is the diameter of the wound made when the experiment started.

The weekly damage increase was estimated by the following formula:

(6) $$Wt_{ILD}=\frac{\left(Diameter\;Wt-Diameter\;D0\right)}{Diameter\;D0}\times 100$$

where *Wt_ILD_* is the increase in leaf damage in any week; *Damage Wt* is the diameter of leaf wound recorded at the end of any given week; *Damage D0* is the diameter of the wound made when the experiment started in any given week.

The corrected insecticidal effect per week of the suspension was calculated using the Schneider–Orelli formula [42]:

(7) $$Corrected\;\%\;insecticidal\;effect\;per\;week=\frac{\left(Mort\;\%\;in\;treated-Mort\;\%\;in\;control\right)}{100-Mort\;\%\;in\;control}\times 100$$

where, *Mort %* is the percentage of aphid cadavers recorded in any given week.

The corrected antifungal efficacy of suspension per week was calculated using the Henderson and Tilton formula ([43] with a few modifications).

(8) $$Weekly\;corrected\;\%\;antifungal\;effect=\frac{\left(Diameter\;\%\;in\;control-Diameter\;\%\;in\;treated\right)}{Diameter\;\%\;in\;control}\times 100$$

where, *Damage* % is the percentage of damage recorded in any given week.

Weekly adjusted mean values by plant were estimated using data from the single CFU, density of conidia in the washed water per leaf surface, the number of leaf adhered spores, mortality, and the damage increase.

### 2.12. Data Analyses

The treatments (2 tents) were not replicated. Thus, whilst undertaking the statistical analyses, the assumption is being made that the between plant variation within each tent is similar to the between plant variation between tents.

A Student–Newman–Keuls (SNK) test was used to compare weekly weather data at *p <* 0.05 using the software GenStat Discovery Edition 4 [44].

For the antifungal experiment, where effects of both treatments in ILD over time were investigated, a longitudinal approach through linear mixed effects models (LMEM) was used with the package *nlme* [45]. The fixed factors were the treatment, week, and the data daily collected (nested in the week of experiment), while the random factor was the plant. Likewise, to test aphicidal activity of the suspension based on the mortality rate (Mt); the same modeling was performed as for the antifungal assessment. However, generalized linear mixed effects models (GLMEM) were specifically used with a Beta family, as data were transformed in percentage for the structure of errors through the packages *MASS* [46] and *glmmTMB* [47]. For different models, a simplification of the fixed terms was performed. The best model was selected based on the Akaike Information Criterion (AIC) using the package *bbmle* [48]. Therefore, the best model was that with the smallest value of AIC. The intra-class correlation (ICC) was computed for the random parts and time variables for the longitudinal models. The adjusted means were estimated for the significant terms of the models. Additionally, a multiple comparison Tukey test was performed to separate means Mt, ILD, and duration between treated and controlled leaves using the package *emmeans* [49]. Means were represented by graphs using the package *ggplot2* [50]. All the analyses for both the antifungal and insecticidal studies were performed at a significance level of α=5 %. in R, version 3.6.1 [51]. Data were in percentage, but they were checked out for normal distribution. Any other transformation was not needed to perform current statistical analyses.

Simple linear regression analyses were run to estimate insecticidal and antifungal activities of the fungal is olate during successive weeks, while Pearson matrix correlation coefficients were estimated between weekly biocontrol efficacy of the entomopathogenic fungus and meteorological data (RH and air temperature). To complete the correlation test, corrected Mt and ILD data were used [42,43]. The software Microsoft XLSTAT 2018 was used in these analyses and tests.

## 3. Results

### 3.1. Weather Patterns

**Figure 3:**
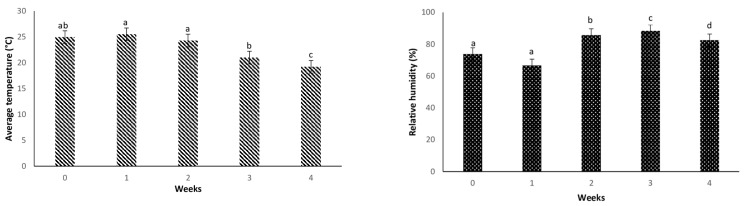
Ambient air temperature and relative humidity data recorded weekly every 30 min. Means followed by different letters are significantly different (*p <* 0.05) between weeks using the SNK test.

Figure 3 summarizes weekly weather data recorded in the plastic house from the suspension spraying until the end of the assessments. Average air temperature and RH showed significant differences (*p* < 0.05) between weeks. Average weekly temperature decreased from week 2–4 (25.52 ± 0.66 °C; 19.23 ± 8.22 °C). However, RH increased from week 1–3 (66.61 ± 1.55%; 88.22 ± 0.9%) and decreased to 82.4 ± 0.69% during week 4 (Figure 3).

### 3.2. Weekly Ectophytic Presence of I. Javanica pf185 after Treatment

Table 1 shows on a weekly basis the persistence of entomopathogenic fungus on the leaf surface of fungus-treated plants.

In the five weeks, fungal spores were present on the leaf blade and ranged from 5.88% (Week 3) to 100% (Week 4) of all white colonies micromorphologically observed after culture. The frequency of colonization of the entomopathogenic fungus compared with the white colonies present after leaf isolation varied with week.

There was a significant difference between fungus-treated and control leaves for the number of colony-forming units. The number of CFUs recorded per mm^2^ of leaf area ranged from 1.1 ± 0.5 to 17.7 ± 6.2 on fungus-treated leaves versus 0.2 ± 0.1 to 9.3 ± 4.0 on control leaves. Similarly, their number varied according to week.

There was a significant difference between fungus-treated and control leaves for the number of spores remaining adhered to the leaves after the vortex running. The number of leaf-adhered spores recorded per mm^2^ of leaf area ranged from 54.5 ± 6.9 to 212.5 ± 9.3 on fungus-treated leaves versus 38.8 ± 5.9 to 137.5 ± 13.7 on control leaves. Similarly, this number varied according to the different weeks covered by the experiment.

There was a significant difference between fungus-treated and controlled leaves for w_w/mm^2^ density after the vortex operation. The density of spores recorded per mm^2^ of leaf area after the vortex ranged from 501.5 ± 38.2 to 964.5 ± 28.5 on fungus-treated leaves versus 192.9 ± 36.1 to 463.0 ± 50.2 on control leaves. Similarly, this number varied according to the different weeks covered by the experiment.

### 3.3. Daily Based Study of Insecticidal Activities on Fungus-Treated and Controlled Leaves per Week

Figure 2 and Table 2 show significant weekly and daily mortality differences in aphid between fungus-treated and controlled leaves (*p* < 0.0001) with a covariance week: treatment (*p* < 0.05).

In addition, plants and weeks explained less of the total variation in aphid mortality with ICC = 0.67% and ICC = 6.5%, respectively (results not shown here).

**Figure 4:**
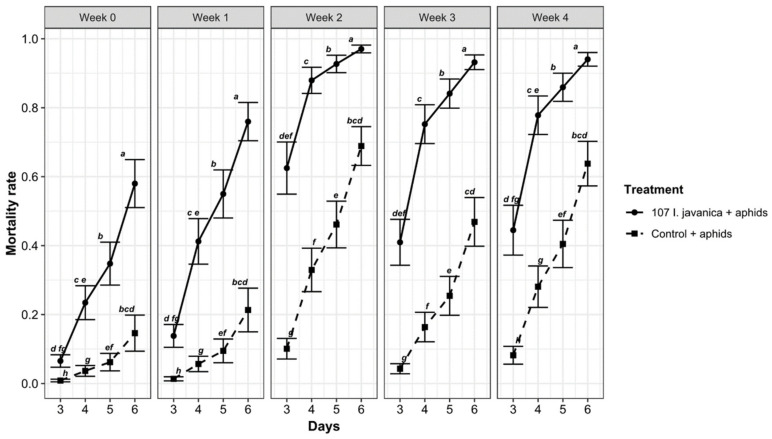
Change in mortality rate across weeks of the experiment for each treatment. Mortality rate: percent of dead aphids after spraying. Days: number of days after spraying. Means with the same letters (a, b, c, d, e, f, g, h) do not differ significantly.

The highest aphid mortality was recorded on fungus-treated leaves, and it increased over weeks and days (Figure 4). The peak value of aphicidal activity was recorded during week 2 (Table 2).

### 3.4. Daily Based Study of the Antifungal Activities on Fungus-Treated and Controlled Leaves per Week

**Figure 5:**
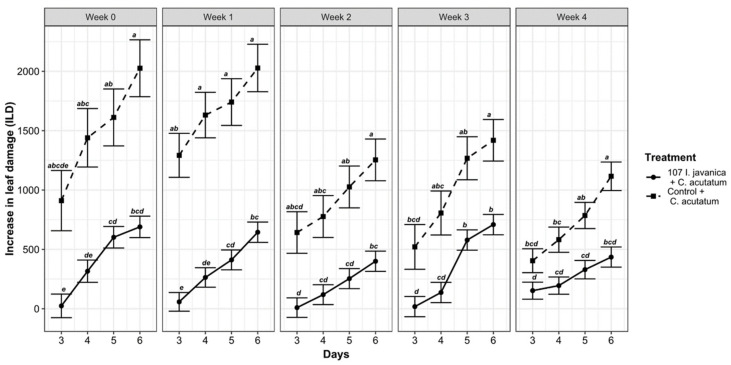
Weekly evolution of the ILD rate per treatment during the experiment. Means with the same letters (a, b, c, d, e, f, g, h) do not differ significantly.

Figure 5 and Table 3 show significant differences between fungus-treated and control plants in ILD over weeks and days (numDF = 1; denDF = 700; F-value = 48.80642; *p* < 0.001).

Plants and weeks explained the total variation in ILD recorded with ICC = 1.43% and ICC = 3.43%, respectively (results not shown here).

The highest ILD was recorded on control leaves and increased weekly and daily (Figure 5). The highest antifungal activity was recorded during week 2 with low ILD value (Table 3).

### 3.5. Suspension Biocontrol Efficacy Per Week

Results revealed W2, W3 and W4 as the periods when the suspension had the most control over pests and disease. The suspension gradually became efficient about two weeks after spraying for both insecticidal and antifungal activities with respect to data recorded at the beginning on W0 assessments (Table 4).

However, by simultaneously exploiting mortality rates recorded during five successive weeks, a regression function of y = 12.583x + 28.642 with a coefficient of determination equivalent to r^2^ = 0.763 was obtained, where y is the mortality rate in a given week and x is the number of weeks.

Similarly, based on antifungal effects on leaves per week during five successive weeks, the regression function of type y = 2.255 x + 33.154 with a coefficient of determination r^2^ = 0.1079 was obtained, where y is the percentage of leaf damage recorded in a given week and x is the entry number of weeks tested.

### 3.6. Weekly Based Study of Correlations Between Biocontrol Efficacy and Weather Patterns

Data analysis showed a strong and positive correlation between insecticidal effect and RH; and a negative one between insecticidal effect and temperature (Table 5). A weak positive correlation was found between fungicidal activity of the entomopathogenic *I. javanica* pf185 and RH. However, no strong correlation was exhibited between fungicidal activity and temperature during the successive assessments (Table 5).

### 3.7. Endophytic Fungi Weekly Isolated from Leaf Tissues of Treated Plants

Culturing of the leaf fragments showed that they hosted a lot of endophytic fungi that belong to several taxonomic groups. Some genera were identified by microscopic observation while others did not (designated as unknown). The entomopathogenic *I. javanica* pf185, which suspension was sprayed, was the most abundant except for W4. From the week of its treatment, this fungus was detected in all treated leaves; but percent of colonization relatively decreased during the following weeks. A virtual absence of this strain was detected on some plants after isolation (Table 6).

**Figure 6:**
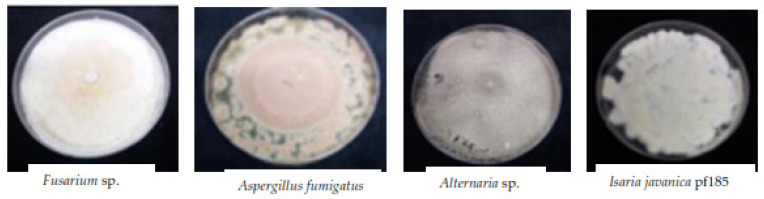
Some taxonomic groups isolated from leaf tissue of fungus-treated plants.

Figure 6 shows a few endophytic taxonomic groups isolated from the leaf tissues of seedlings sprayed during successive studies.

## 4. Discussion

These experiments were carried out to test the insecticidal and fungicidal potential of the entomopathogenic fungus *I. javanica* pf185 focused on short-term (successive weeks) protection against the green peach aphid (*M. persicae*) and anthracnose (*C. acutatum*) in pepper plants. To our knowledge, this is the first study to continuously document biological control activities of this endophytic fungus in the tested crop. It also concentrated on the persistence of spores of the entomopathogen outside and inside leaves after spraying, followed by micromorphological identification of different taxonomic groups present among the isolated white mycelia.

There was a significant difference in the number of CFUs and the concentration of the solution obtained after dilution (density w_w) per square millimeter of sprayed leaves, and it varied according to week. The five different weeks of the experiment were characterized by particular climatic conditions (Figure 3). The majority of other species such as *Aspergillus fumigatus*, *Cladosporium sphaerospermum,* and *Verticillium* sp. appeared to reduce the frequency of *I. javanica* pf185 in cultured leaf tissue (Table 6). In addition, Table 5 shows the correlation between certain climatic factors and the activity of the entomopathogenic fungus, but Table 6 shows the diversity of fungal species that can be observed after isolation. These results corroborate previous work from other researchers who showed that the diversity of microorganisms (fungi and bacteria) observed on the surface and inside plants depends on several factors. For example, Karlsson et al. [52] mentioned the influence of chemical fungicide on microorganism colonies on the leaves of wheat. Similarly, many studies revealed that several phyllosphere fungal communities associated with wheat varied with the seasons [53,54]. Fungi can be present both as epiphytes and endophytes on pepper leaves is consistent with the successive weekly studies conducted by culturing leaf wash liquid and leaf sterilized surface pieces. This variation in fungal communities is consistent with previous findings on wheat [55]. The main components of the fungal community differed in these studies conducted during successive weeks and the mechanisms. Therefore, the dynamics of fungal communities on phyllosphere of the crops seem poorly understood. Aside from the presence of several fungal communities, this study demonstrated that the released fungal isolate had colonized foliar tissues. This was confirmed by successive micromorphological isolations and identifications. It also revealed that endophytic fungus was established and still present on the leaf surface.

The insecticidal activity study showed a significant difference between fungus-treated leaves and controlled ones (Table 3). However, the mortality rate recorded on the fungus-treated leaves constantly varied over the days to reach its maximum on D6. These results provide information on the virulence of the endophytic fungus, which was found on the first dead insects about three days after inoculation. A conclusion could already be drawn on D4, except during W4 where the mortality recorded on D5 and D6 was significantly different to that on D3 and D4. After the spray of suspension on plants, the period of action called gauge of the endophytic efficacy depends on hosts and environmental conditions, which are correlated with fungal activities [56,57]. The assessment method almost reflected how the endophytic isolate can behave under field conditions. The insects were thus placed onto the leaf discs in the laboratory to simulate the field results. This confirmed findings of Kang et al. [32], which focused on the same organisms, both the fungal isolate and the aphid species, during only one week. These authors mentioned that its efficacy is closely related to the concentration of suspension and the number of days after spraying. Despite differences in the duration of these studies, our conclusions were quite similar. These results reinforce the biocontrol effectiveness of the fungus during W2, which recorded the highest mortality and the lowest ILD rates compared to other weeks. Due to the high mortality and the lowest ILD recorded in W2, characterized by a temperature of about 25 °C and an RH above 80%, it appears that climatic factors impacted the performance of *I. javanica* (strain pf185). The highest mortality noticed from W2 confirmed previous findings in aphids where this same fungus isolate was used [32]. Those authors mentioned that the number of dead aphids after spraying of suspensions at 10^6^, 10^8^ and 10^9^ conidia/mL significantly increased accordingly. Mortality appeared in plants treated with 10^8^ conidia/mL, while the average recorded at 10^6^ conidia/mL was about 10% on the 6th day after treatment. However, over 90% mortality was recorded six days after spraying of 10^9^ conidia/mL. To achieve aphid mortality, the microorganism must adhere to the cuticle of its host, penetrate it using specific enzymes, and begin its life cycle inside the host body. These successive phenomena lead to death of the insect a few days later [58]. Luo [58] earlier showed that efficacy of *Metarhizium acridum* in the presence of macrophages; aphid mortalities recorded on fungus-treated leaves may also result from the effects of direct contact with the fungus or the activity of synthetic products in plant tissues [59,60]. The effectiveness of a microorganism for biological control depends on its ability to penetrate the tissues of its host, but also depends on its ability to biosynthesize secondary metabolites and volatile organic compounds (VOCs) that intoxicate or inhibit the development of plant enemies such as arthropods and pathogens among others [61,62]. The development of secondary metabolites and VOCs is a medium and long-term process that results from a symbiotic relationship between endophytic microorganisms, plants and abiotic factors [63,64]. Other studies on endophytic fungi revealed the presence of several bioactive natural products which possess significantly higher anticancer, insecticidal, and antimicrobial potentials [65]. During this study, the mortality rates noted from W0 to W4 can be closely associated with the development of secondary metabolites and other specific functions existing in the entomopathogenic fungus, but unfortunately, our study did not focus on its biochemical contents. However, Lee et al. 62] showed that the secondary metabolite dibutyl succinate, elaborated by *I. javanica* pf185, is active against aphids. This previous molecule, which accumulates in the plant tissues, could have acted on the aphids during this experiment reducing their populations.

The daily mean damage per leaf was higher on control leaves versus fungus-treated ones. The comparison of damage recorded in fungus-treated leaves during consecutive days also showed a substantial reduction in damage. Similarly, a weekly comparison between fungus-treated and controlled ones showed a reduction in amount of damage recorded on fungus-treated leaves. These results confirmed those obtained during the daily assessment; more damage occurred as the weeks progressed, but relatively lower than the previous week one. The results in Table 2 and Table 3 give a relative antifungal capacity in *I. javanica* pf185 against anthracnose transmitted by *C. acutatum*. Likewise, Lee [66], regarding the same entomopathogenic isolate, revealed the fungicidal action of both supernatant and its secondary metabolite dibutyl succinate on anthracnose caused by *C. acutatum*. However, their seven-day assessment only showed a weak efficacy of the compound against the pathogenic fungus under laboratory conditions. Similarly, the findings of Kang [32] showed the antagonism between *C. gloeosporioides* and *I. javanica* strains by testing them on a culture medium for seven days. Additionally, these previous results confirmed the inhibitory action of *I. javanica* on the development of *C. gloeosporioides* during a seven-day assessment period. It appears a complex mechanism to optimize the use of endophytes for an in-depth understanding interaction between endophyte, host, and plant pathogen. This includes competition for space and nutrients, direct inhibition through antibiosis, mycoparasitism, and induced resistance in the plant by activating its own defense system among other factors [67,68,69]. This phenomenon is governed by a multitude of parameters; therefore, we essentially focused on damage increase to prevent speculation.

In addition, the biocontrol efficacy of *I. javanica* was revealed by insecticidal and antifungal data in Table 4. These results showed an ability of *I. javanica* strain pf185 to control aphids than anthracnose due to *C. acutatum* under the experimental conditions. Thus, W2, W3, and W4 showed the ability of entomopathogenic fungus to better control aphids and anthracnose during the assessments. The technical implication of the regression function, mortality, and damage with weeks after inoculation relative to the experimental conditions, shows that an aphid infestation can be eradicated by a suspension at 10^7^ conidia/mL during 5.67 weeks, while it would take about 29.64 weeks to eliminate the pathogen in those same conditions. The insecticidal efficacy of the suspension should incorporate environmental data such as RH and temperature, which showed a positive and a negative correlation with aphid mortality, respectively (Table 5). These results showed the primordial role of climatic parameters in the efficacy of entomopathogenic fungus revealed by previous studies [70,71]. Tian et al. [70] mentioned that the conidial germination rate of *I. fumosorosea* was higher when RH ranged from 85% to 95%, but lower or no germinated when RH was below 75%. Luz [71] observed that isolates of *M. anisopliae* and *B. bassiana* were highly virulent to *Triatoma infestans* 3rd instar nymphs at an RH > 98% and a temperature of 25 ± 0.5 °C. During that same study, results showed that biocontrol activity against nymphs of *T. infestans* was reduced at 75% RH. Moreover, the strong negative correlation found between the temperature and the insecticidal effect of the fungus shows the key role played by this factor in the endophyte’s efficacy. Its role was previously mentioned by several researchers who showed a temperature gap beyond it becomes harmful to the effectiveness of certain fungi [34,72]. However, the study revealed a reduction in diameter of the symptoms on the leaf, confirming prior results on *C. Acutatum* mycelial growth using *I. javanica* pf185 [66]. It is documented that IPM is the most important tool to sustainably keep fungal threats below the economic threshold, but several mechanisms participate to the control of crop enemies using endophytic fungi [73].

## 5. Conclusions

Overall, this work has shown the insecticidal and fungicidal activities of the entomopathogenic fungus *I. javanica* pf185. It revealed a strong positive correlation between insecticide efficiency and weather patterns. Therefore, no correlation was found between antifungal efficiency against *C. acutatum* and weather patterns. Based on the bioassays on *M. persicae* and *C. acutatum*, it would be advisable to conduct metabolic studies to identify the effects of VOCs and metabolites involved in the biological control process. It would also be important to test in vitro the impact of weather parameters on growth and infectivity of the entomopathogenic isolate. The next step of our activities is to determine all metabolic compounds present in this isolate. A later study involving the individual role of each/all the compounds, in combination is necessary, that would help to broaden knowledge on biological activities of *I. javanica* pf185.

## Figures and Tables

**Table 1:** Weekly ectophytic persistence of *I. javanica*; and evaluation of total CFUs and adhered spore density of washed spores; and pf185 after spraying.

Weeks	%Ecto *I. javanica*	CFU/mm^2^		Adhered Spores/mm^2^		Density w_w/mm^2^	
		Control	Treated	Control	Treated	Control	Treated
**0**	23.26	0.5 ± 0.2 b	2.7 ± 0.6 a	101.4 ± 12.7 b	212.5 ± 9.3 a	192.9 ± 36.1 b	964.5 ± 28.5 a
1	46.51	0.2 ± 0.1 a	1.1 ± 0.5 a	99.0 ± 6.0 a	117.7 ± 10.3 a	231.5 ± 28.5 b	501.5 ± 38.2 a
2	20.93	4.9 ± 2.0 b	17.1 ± 6.2 a	38.8 ± 5.9 a	54.5 ± 6.9 a	424.4 ± 32.5 b	733.0 ± 36.1 a
3	4.65	9.3 ±4.0 a	10.5 ± 2.0 a	137.5 ± 13.7 b	180.7 ± 12.1 a	347.2 ± 27.0 a	578.7 ± 46.8 a
4	4.65	5.6 ± 1.5 b	16.6 ± 4.2 a	98.0 ± 8.3 a	117.2 ± 11.1 a	463.0 ± 50.2 a	733.0 ± 46.8 a

CFU: colony-forming units. Treated: fungus-treated. ±: Standard error value. %Ecto *I. javanica*: percent of *I. javanica* pf185 found among the white ectophytic fungi. For the same parameter, means for control and fungus-treated leaves followed by different letters are significantly different (*p* < 0.05).

**Table 2:** Weekly mortality per treatment during assessment of aphicidal activity.

Weeks	Treatment	Mean (%)	SE	CV	Min	Max
0.	*I. javanica* pf185 + Aphid	25.56	4.08	135.37	0.00	100.00
0	Control + Aphid	8.61	1.47	144.95	0.00	40.00
1	*I. javanica* pf185 + Aphid	42.78	4.48	88.88	0.00	100.00
1	Control + Aphid	11.94	2.23	158.13	0.00	80.00
2	*I. javanica* pf185 + Aphid	83.33	3.04	30.94	0.00	100.00
2	Control + Aphid	40.56	2.56	53.62	0.00	100.00
3	*I. javanica* pf185 + Aphid	73.33	3.47	40.17	0.00	100.00
3	Control + Aphid	22.22	2.33	88.7	0.00	80.00
4	*I. javanica* pf185 + Aphid	80.28	3.27	34.61	0.00	100.00
4	Control + Aphid	28.89	2.85	83.70	0.00	80.00

SE: Standard error of the mean, CV: Coefficient of Variation, Min: minimum, Max: Maximum.

**Table 3:** Summary of the weekly ILD per treatment during antifungal assessment.

Weeks	Treatment	Mean (%)	SE	CV	Min	Max
0	*I. javanica* pf185 + *C. acutatum*	525	52.991	85.647	0	1600
0	Control + *C. acutatum*	1173.91	121.998	86.326	200	4800
1	*I. javanica* pf185 + *C. acutatum*	419.44	42.636	86.252	0	1200
1	Control + *C. acutatum*	1500	88.103	49.839	200	3800
2	*I. javanica* pf185 + *C. acutatum*	225	36.673	138.301	0	1200
2	Control + *C. acutatum*	863.89	74.622	73.295	0	3000
3	*I. javanica* pf185 + *C. acutatum*	369.44	48.878	112.261	0	2000
3	Control + *C. acutatum*	983.33	88.965	76.768	0	3400
4	*I. javanica* pf185 + *C. acutatum*	279.37	32.804	93.201	0	1000
4	Control + *C. acutatum*	733.33	56.085	64.895	0	2000

SE: Standard error of the mean, CV: Coefficient of Variation, Min: minimum, Max: maximum.

**Table 4:** Entomopathogenic suspension insecticidal and antifungal efficacies per week.

Weeks	Insecticidal Efficacy (%)	Antifungal Efficacy (%)
0	20.43	26.07
1	39.82	38.01
2	72.32	53.35
3	66.43	29.08
4	70.04	41.81

**Table 5:** Correlation between weekly biocontrol efficacy and weather patterns.

Variables	Insecticidal	Antifungal	Humidity	Temperature
Insecticidal	1			
Antifungal	0.638	1		
Humidity	0.788	0.248	1	
Temperature	−0.651	0.002	−0.648	1

**Table 6:** Endophytic fungi isolated weekly from leaf tissues.

Weeks	Plants	Isolated	*I. javanica* pf185	*Verticillium* sp.	*Alternaria* sp.	Unknown	*Aspergillus* *fumigatus*	*Acremonium* sp.	*Fusarium* sp.	*Cladosporium* *sphaerospermum*
0	TP1	73	73	0	0	0	0	0	0	0
	TP2	53	34	2	2	15	0	0	0	0
	TP3	32	1	0	0	0	31	0	0	0
1	TP1	54	44	0	6	1	0	3	0	0
	TP2	20	20	0	0	0	0	0	0	0
	TP3	51	51	0	0	0	0	0	0	0
2	TP1	30	0	0	0	0	30	0	0	0
	TP2	37	22	2	0	1	8	0	4	0
	TP3	19	1	9	0	0	9	0	0	0
3	TP1	33	15	0	16	0	0	0	0	2
	TP2	35	35	0	0	0	0	0	0	0
	TP3	36	35	0	0	0	0	0	1	0
4	TP1	10	0	0	10	0	0	0	0	0
	TP2	10	0	0	0	0	0	0	0	10
	TP3	14	2	0	0	0	0	12	0	0
Endo CF			65.68	2.56	6.71	3.35	15.38	2.96	0.99	2.37

TP_z_ = Fungal-treated plant entry number z, Unknown= Unidentified fungi. Endo CF = Endophytic colonization frequency, Sp = Species.

## Data Availability

Not applicable.
